# Insights into the role of three Endonuclease III enzymes for oxidative stress resistance in the extremely radiation resistant bacterium *Deinococcus radiodurans*

**DOI:** 10.3389/fmicb.2023.1266785

**Published:** 2023-09-12

**Authors:** Filipe Rollo, Guilherme D. Martins, André G. Gouveia, Solenne Ithurbide, Pascale Servant, Célia V. Romão, Elin Moe

**Affiliations:** ^1^Instituto de Tecnologia Química e Biológica António Xavier, Universidade NOVA de Lisboa, Oeiras, Portugal; ^2^Université Paris-Saclay, CEA, CNRS, Institute for Integrative Biology of the Cell (I2BC), Gif sur Yvette, France; ^3^Department of Chemistry, UiT - The Arctic University of Norway, Tromsø, Norway

**Keywords:** DNA repair, DNA glycosylase, radiation damage, base excision repair, oxidation damage

## Abstract

The extremely radiation and desiccation resistant bacterium *Deinococcus radiodurans* possesses three genes encoding Endonuclease III-like enzymes (DrEndoIII1, DrEndoIII2, DrEndoIII3). *In vitro* enzymatic activity measurements revealed that DrEndoIII2 is the main Endonuclease III in this organism, while DrEndoIII1 and 3 possess unusual and, so far, no detectable EndoIII activity, respectively. In order to understand the role of these enzymes at a cellular level, DrEndoIII knockout mutants were constructed and subjected to various oxidative stress related conditions. The results showed that the mutants are as resistant to ionizing and UV-C radiation as well as H_2_O_2_ exposure as the wild type. However, upon exposure to oxidative stress induced by methyl viologen, the knockout strains were more resistant than the wild type. The difference in resistance may be attributed to the observed upregulation of the EndoIII homologs gene expression upon addition of methyl viologen. In conclusion, our data suggest that all three EndoIII homologs are crucial for cell survival in stress conditions, since the knockout of one of the genes tend to be compensated for by overexpression of the genes encoding the other two.

## 1. Introduction

*Deinococcus* species, especially *Deinococcus radiodurans* has been described as the most radiation resistant bacteria on earth. It can withstand up to 5 kGy of ionizing radiation (IR) with no loss of viability (Moseley and Mattingly, 1971; Battista, 1997). Exposure to such high doses of radiation generates hundreds of double strand breaks (DSBs), thousands of single strand breaks (SBDs) and greater than 1,000 sites of base damage, which *D. radiodurans* can repair within hours (Battista, 1997; White et al., 1999; Slade and Radman, 2011; Daly, 2012). The mechanism of this extreme resistance is not known but generally believed to be due to a combination of factors such as its densely packed genome, high levels of pigments (e.g., deinoxanthin) in the membrane, a high level of intracellular Manganese, and an extremely efficient DNA repair mechanism.

The genome of *D. radiodurans* encodes several DNA glycosylases with homologs in all three domains of life involved in the Base Excision repair (BER) pathway (Makarova et al., 2001; Daly, 2012). Endonuclease III (EndoIII) is a bifunctional DNA glycosylase, belonging to the Helix-hairpin-Helix (HhH)-GDP superfamily of DNA glycosylases, which removes a broad range of oxidized DNA lesions like thymine glycol (Tg) and 5-hydroxycytosine (5OHC) in the BER pathway (Dizdaroglu, 2015; Sarre et al., 2019). The most common oxidized lesions are 5OHC and 5-hydroxyuracil (5OHU) which is generated by oxidation of cytosine and Tg which is generated through either oxidation of thymine or oxidation and deamination of 5-methylcytosine. Abasic sites (AP-sites) is caused by loss of a nucleobase, and dihydrothymine (DHT) and dihydrouracil (DHU) originate from IR damage of thymine and cytosine (Dizdaroglu, 1985; Breen and Murphy, 1995). The main substrate of EndoIII enzymes is Tg. This lesion is not mutagenic, but cytotoxic and has been shown to affect cell division by blocking DNA polymerases due to the perturbation induced in the DNA structure (Ide et al., 1985; Clark and Beardsley, 1986; Wallace, 2002; Dizdaroglu, 2015).

In chromosome I, *D. radiodurans* possesses three genes encoding for EndoIII-like glycosylases DR2438, DR0289 and DR0928 (*endoIII1*, *endoIII2* and *endoIII3,* respectively). The gene products of *endoIII1* and *endoIII3* (DrEndoIII1 and DrEndoIII3) were initially considered to be of archaeal origin, while *endoIII2* (DrEndoIII2) was found to be closest to yeast EndoIII (Makarova et al., 2001). Homologs of these three enzymes have been found in other *Deinococcus* species except in *D. peraridilitoris* which lacks the *endoIII3* (Lim et al., 2019; Sarre et al., 2019). Later, sequence alignments of the three EndoIII enzymes with homologs found in other bacterial, archaeal and eukaryotic species led to the hypothesis that these enzymes could be of bacterial origin and that DrEndoIII3 is specific to the *Deinococcaceae* genus (Sarre et al., 2015).

Structure determination of DrEndoIII1 and 3, and homology modelling of DrEndoIII2 revealed that they consist of two α-helical domains (A and B) divided by a positively charged DNA binding cleft, which contains the highly conserved HhH motif and the catalytic residues (aspartate and lysine). Domain A harbors a [4Fe-4S] cluster and a FeS cluster loop and domain B two DNA intercalating loops (DIL1 and DIL2) which are involved in the stabilization of DNA when the damaged base is flipped out of the DNA into the active site pocket. Even though all DrEndoIII homologs can bind undamaged and damaged DNA with various affinities, only DrEndoIII1 and DrEndoIII2 demonstrated activity towards the common EndoIII substrates (Sarre et al., 2015, 2019). DrEndoIII2 revealed catalytic properties that exceed those of *E. coli* EndoIII (EcEndoIII), while DrEndoIII1 acts mostly as a monofunctional DNA glycosylase with lower activity, being also able to process single stranded DNA (ssDNA) (Sarre et al., 2019). Mutational studies have been conducted to unravel the catalytic differences between these enzymes. Substitutions introduced in DIL1 and DIL2 of DrEndoIII1, revealed that these mutations led to a decreased activity due to a lower substrate affinity (Rollo et al., 2022). In the case of DrEndoIII3, none of the substitutions performed in the active site pocket led to an induction of EndoIII type activity (Rollo et al., 2022).

To unravel the role of these three enzymes in the repair of oxidative damages, transcriptome profiling and EndoIII knockout (KO) mutants have been generated and analyzed (Liu et al., 2003; Chen et al., 2007; Hua et al., 2012). However, several questions remain to be answered from these studies. Cells irradiated with a total dose of 15 kGy revealed a down regulation of *endoIII1* with no alterations of the expression of *endoIII2* and *endoIII3* (Liu et al., 2003). On the other hand, only *endoIII1* and *endoIII3* were observed to be damage activated, by gene up regulation, immediately after exposure to 2 kGy of IR (Chen et al., 2007). DrEndoIII KO mutants (Hua et al., 2012) showed no significant difference between the mutants and wild type (WT) when exposed to both IR and hydrogen peroxide (H_2_O_2_). This has also been observed in EndoIII KO mutant in *E. coli,* which was shown to be as resistant as the WT (Cunningham and Weiss, 1985). Moreover, in *D. radiodurans* each EndoIII KO mutant showed elevated levels of spontaneous mutation which were increased in the triple KO mutant leading to the hypothesis that the three EndoIII’s are not only required to repair DNA damage but also have non-overlapping substrates (Hua et al., 2012).

To shed new light on the role of these enzymes at a cellular level in *D. radiodurans*, *endoIII1*, *endoIII2,* and *endoIII3* single KO mutants were constructed and analyzed after being subjected to various oxidative stress related conditions. Here we report the characterization of these strains in terms of their resistance to ionizing and UV-C radiation and exposure to H_2_O_2_ and methyl viologen (MV) (also known as the common herbicide Paraquat). In the latter case, the effect of stress on the expression level of the *endoIII* genes was also analyzed. Our data suggests that the KO mutants are as resistant as the WT to IR, UV-C and H_2_O_2_ exposure, however upon exposure to MV they were revealed to be more resistant than the WT which could be attributed to the observed upregulation of EndoIII homologs gene expression levels. Due to an increasing amount of *Deinococcus* sequenced genomes, we also performed a phylogenetic analysis in order to increase our insight regarding the origin of the genes.

## 2. Materials and methods

### 2.1. Phylogenetic analysis

A phylogenetic analysis was carried out based on the alignment of 100 EndoIII protein sequences from 17 genome sequenced *Deinococcus* species (*D. radiodurans, D. geothermalis, D. deserti, D. maricopensis, D. proteolyticus, D. gobiensis, D. peraridilitoris, D. swensis, D. soli, D. actinosclerus, D. puniceus, D. irradiatisoli, D. wulumuqiensis, D. ficus, D. grandis, D. psychrotolerans, D. radiophilus*) as well as representative EndoIII proteins from *Homo sapiens, Mus musculus, Xenopus tropicalis, Drosophila melanogaster, Danio rerio, Arabidopsis thaliana, Escherichia coli, Saccharomyces cerevisiae, Caenorhabditis elegans, Bacillus subtilis, Mycobacterium tuberculosis, Chroococcidiopsis thermalis, Thermococcus gammatolerans, Pyrobaculum aerophilum,* and *Archaeoglobus fulgidus*. Proteins aligned by ClustalW (Thompson et al., 1994) were used for development of a maximum likelihood phylogenetic tree with the WAG+G (Whelan and Goldman, 2001) model in MEGAX (Kumar et al., 2018). For calculation of the bootstrap values 1,000 replications were performed. The phylogenetic tree was visualized using iTOL (Letunic and Bork, 2021).

### 2.2. Bacterial strains, media, growth conditions and construction of *Deinococcus radiodurans* mutants

The *E. coli* strain DH5α was used to propagate plasmids in Luria Broth at 37°C with appropriate antibiotics used at the following concentrations: hygromycin 50 μg/mL, ampicillin 100 μg/mL. All *D. radiodurans* strains were derivatives of strain R1 ATCC 13939, and were grown at 30°C and shaking at 150 rpm in TGY medium [1% (w/v) of Tryptone (VWR), 0.5% (w/v) of Yeast Extract (VWR), 0.5% of Glucose (Carl Roth) 20% (w/v)]. The media were supplemented with the appropriate antibiotics used at the following concentrations: hygromycin 50 μg/mL, kanamycin 6 μg/mL, chloramphenicol 3.5 μg/mL. The GY16076 (∆*dr2438Ωkan, alias* ∆EndoIII1), GY16078 (∆*dr0289*Ω*cat, alias* ∆EndoIII2) and GY16080 (∆*dr0982*Ω*hph, alias* ∆EndoIII3) mutant strains were constructed by replacement of the corresponding locus with the appropriate antibiotic resistance cassette using the tripartite ligation method (Mennecier et al., 2004). The transformation of *D. radiodurans* with PCR products was performed as previously described (Bonacossa de Almeida et al., 2002). p11086, p12625 and pPS6 plasmids (Meyer et al., 2018) were used as template to amplify kanamycin, hygromycin and chloramphenicol resistance genes, respectively.

### 2.3. DNA manipulations

Plasmid DNA was extracted from *E. coli* using the NucleoSpin® Plasmid miniprep kit (Macherey-Nagel). *D. radiodurans* chromosomal DNA was isolated as described previously (Norais et al., 2013). PCR reactions were carried out with Phusion DNA Polymerase (Thermo Scientific) to amplify fragments subsequently used for cloning, or with GoTaq Flexi DNA Polymerase (Promega) for diagnostic PCR. PCR products were purified using the PCR Clean-up kit (Macherey-Nagel). All the oligonucleotides used for the knockout mutant constructions are listed in Table 1.

**Table 1:** List of the primers used in the generation of knockout mutants.

Name	Sequence 5′-3′
EndoIII1 Up FP (SI78)	GCAGGAAGAACATGCCAAAG
EndoIII1 Up RP (SI79)	CTGATCTAGAAGCGAGAAACCGGTCAAAGG
EndoIII1 Kan FP (PS39BamHI)	GGAATCTAGAGCAAGCAGCAGATTACG
EndoIII1 Kan RP (PS178XbaI)	GGAAGGATCCGCATTCTGCTCCAGCATCTC
EndoIII1 Down FP (SI80XbaI)	ATTCGGATCCACACGTCGCCGAAGAGGGTC
EndoIII1 Down RP (SI81)	TCGAACGCCCGGTCAAACTC
EndoIII2 Up FP (SI78)	CGGCGGCGGGCAGTCCCAAG
EndoIII2 Up RP/SI79BamHI	GCTAGGATCCCTAGGCCAGAAGGCTTAGTG
EndoIII2 CAM FP/PS39BamHI	ACGGGATCCCTTTGGAACGGTGCTCGGTG
EndoIII2 CAM RP/PS178XbaI	ATTTCTAGACGCGGCCGCACTTATTCA
EndoIII2 Down FP/SI80XbaI	ATGCTCTAGAGGTGGAGCATGTCGAGGGTT
EndoIII2 Down RP (SI81)	GTCATCAGGGCGCTTTGCAC
EndoIII3 FP (SI90)	CCGTTGACCACGCCGATTTG
EndoIII3 Up RP (SI91Agel)	GACAACCGGTAGGGCACAGGGCAGCGTAAC
EndoIII3 Hph FP (SI97Agel)	TCAGACCGGTGCTTGATATCGAATTCGAGC
EndoIII3 Hph RP (EB87XbaI)	AATTCTAGAGATCCGTGTTTCAGTTAGCC
EndoIII3 down FP (SI92XbaI)	GTACTCTAGAAGCCGTCCGAGTTGGAGTGG
EndoIII3 down RP (SI93)	ACTCGTTTCCGGACACGTTC

The restriction sites used were underlined.

### 2.4. Sensitivity assays to γ-radiation

Cultured cells in the exponential growth phase were concentrated to an A_650nm_ = 20 in TGY2X and irradiated on ice with a ^137^Cs irradiation system (Institut Curie, Orsay, France) at a dose rate of 41.8 Gy/min. Following irradiation, diluted samples were plated on TGY plates. Colonies were counted after 3–4 days incubation at 30°C.

### 2.5. Sensitivity assays to UV-C radiation

*D. radiodurans* strains grown in TGY to an optical density (OD_600nm_) of 2.0 were sequentially diluted and plated on TGY medium. 5 μL drops were air dried and exposed to final doses between 900 J/m^2^ and 1800 J/m2 of UV-C radiation (254 nm). A fluence rate of 3 mW/cm^2^ was checked using a MS-100 optical radiometer equipped with an MS 125 UV-C sensor (Ultra-Violet Products, Upland, CA). Each independent assay consisted of two technical replicates within each biological replicate. After irradiation, the cells were incubated at 30°C for 3 days. Cell survival was assessed by measuring the colony-forming units (CFU) at any given dose (N) and dividing it by the control conditions (N_0_), (which were not exposed to radiation). Cell survival curves were obtained by plotting the logarithm of (N/N_0_) and dose (J/m^2^).

### 2.6. Sensitivity assays to H_2_O_2_ and MV

The resistance of *D. radiodurans* to H_2_O_2_ and MV oxidative stress was assessed by adapting the Kirby Bauer method (Holder, 1989). Cells were grown to an OD_600nm_ of 2.0 and a total of 400 μL were swabbed evenly in a 120×120 mm plate (VWR) and air-dried for at least 15 min. Afterwards, 6 mm disks (Prat Dumas) were placed on top of the bacterial mat, and 20 μL of increasing concentrations of either H_2_O_2_ (0.2 to 1 M) or MV (WT, 2 to 10 mM while ∆EndoIII strains 10 to 50 mM) were applied. MilliQ H_2_O was used for the control conditions and drops were air-dried. Plates were incubated at 30°C for 3 days and oxidative stress response was assessed by measuring the diameter of the inhibition zones using ImageJ (Schneider et al., 2012). Two independent assays with three replicates were performed.

### 2.7. Effects of MV-induced oxidative stress during cell growth

*D. radiodurans* strains were grown in TGY medium with the appropriate antibiotics, at 30°C, and 150 rpm. The effect of exposure to oxidative stress was tested by adding 0.1 mM MV to the culture flasks. This concentration was chosen based on initial growth experiments in liquid media with different concentrations of MV and is lower than in the sensitivity assays which were performed on solid media. The compound was added to the media when the cell cultures had obtained an optical density (OD_600nm_) of 0.3. Growth curves of each strain in absence and presence of MV were generated from three independent growths, and evaluated by plotting OD_600nm_ against the incubation time, followed by an analysis of the gene expression at the mRNA level at different time points in the growth to analyse the expression profile of the different strains in both conditions.

### 2.8. RNA extraction cDNA synthesis and RT-PCR

Total RNA was extracted from cells which were harvested at different time points (0 h (before the addition of 0.1 mM MV (OD_600nm_ = 0.3)), 2 h (early exponential phase) and 5 h (mid exponential phase)) by using the RNAspin Mini RNA isolation kit (Cytiva, Buckinghamshire, UK) with some modifications. 5 mL of cultured *D. radiodurans* cells were harvested by centrifugation at 4°C, 11000 *g* for 5 min, and resuspended in 100 μL of Tris-EDTA (TE) buffer (10 mM Tris–HCl pH 8, 1 mM EDTA) containing 45 mg/mL lysozyme followed by 3 min of vigorous vortexing and incubation for 45 min at 37°C. After incubation, the total RNA extraction was performed according to the manufacturer’s instruction. The RNA was quantified and tested for purity by calculating the A_260_/A_280_ ratio using a spectrophotometer (Nanodrop) and gel quantification. Extracted RNA with a purity of 1.8–2.0 was used for cDNA synthesis. The cDNA synthesis was performed using 2 μg DNase treated RNA and the Transcriptor High Fidelity cDNA Synthesis Kit (Roche, Basel, Switzerland) according to the manufacturer’s instructions.

For the RT-PCR, primers were designed for each gene of interest, *endoIII1*, *endoIII2* and *endoIII3,* and for two housekeeping genes (Glyceraldehyde 3-phosphate dehydrogenase (*GAPDH*) and DNA gyrase A (*gyrA*)) (Table 2) of *D. radiodurans* and checked for the absence of self-complementarity and gene selectivity. Gene expression levels were determined from a gel based semi-quantitative analysis and normalized against the housekeeping gene expression levels. RT-PCR products were loaded onto 1% TBE (100 mM Tris–HCl, 100 mM Boric acid, 2 mM EDTA) agarose gel and bands were visualized on a Fuji TLA-5100 scanner using a LPB filter (473 nm). Band quantification was performed using Fiji (Schindelin et al., 2012). The data presented for each gene is from three biological replicates and the gene expression levels were subjected to two-way ANOVA tests to detect significant (*p* < 0.0332) differences between expression levels.

**Table 2:** List of the primers used in the RT-PCR for gene expression levels analysis.

Name	Sequence 5′-3′
DrEndoIII1 RT-PCR FP	CGTATCTGTACGAGCTGCACATCA
DrEndoIII1 RT-PCR RP	TATCCTTTAGCCGGTTTCTCGCCT
DrEndoIII2 RT-PCR FP	ACAAAGTGGAAGCCGACCTGCAAA
DrEndoIII2 RT-PCR RP	TACTTTCGGACAGAAGCTCGCCAA
DrEndoIII3 RT-PCR FP	ACACTGAGCAGGATGGAGAATGGA
DrEndoIII3 RT-PCR RP	TGTTCGCTCAGAGGACGGGATTTA
DrGAPDH RT-PCR FP	AGGAAGTCAACAACGTCTTCCGTG
DrGAPDH RT-PCR RP	TACCACGAGAAGAACTTGACGAGG
DrgyrA RT-PCR FP	TCAGACCATCAGCGCGATGTACAA
DrgyrA RT-PCR RP	CTGGGTGTACTTGTAGAGCTGGTT

## 3. Results

### 3.1. Phylogenetic relationship of the DrEndoIII homologs

**Figure 1:**
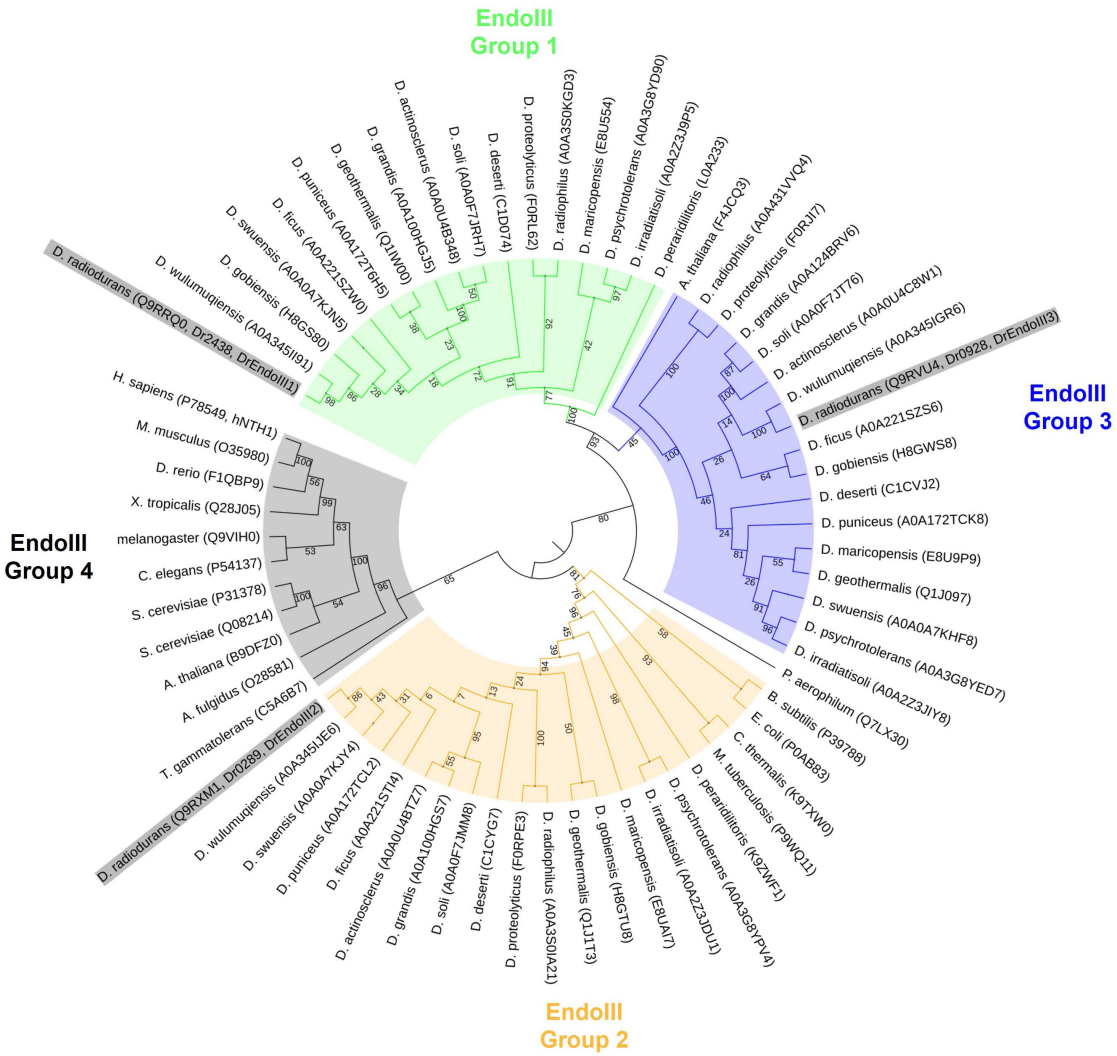
Maximum likelihood phylogenetic tree using model WAG+G in MegaX. Four groups of EndoIII family proteins were identified from 17 *Deinococcus* species (*D. radiodurans, D. geothermalis, D. deserti, D. maricopensis, D. proteolyticus, D. gobiensis, D. peraridilitoris, D. swensis, D. soli, D. actinosclerus, D. puniceus, D. irradiatisoli, D. wulumuqiensis, D. ficus, D. grandis, D. psychrotolerans, D. radiophilus*) and other organisms generally considered to be model organisms (*Homo sapiens, Mus musculus, Xenopus tropicalis, Drosophila melanogaster, Danio rerio, Arabidopsis thaliana, Escherichia coli, Saccharomyces cerevisiae, Caenorhabditis elegans, Bacillus subtilis, Mycobacterium tuberculosis, Chroococcidiopsis thermalis, Thermococcus gammatolerans, Pyrobaculum aerophilum* and *Archaeoglobus fulgidus*). Uniprot accession codes of each protein are included in parentheses following the species name. The node numbers are bootstrap values based on 1,000 replicates. DrEndoIII’s are highlighted in grey in the figure.

Due to an increasing amount of recently sequenced *Deinococcus* strains, we performed a phylogenetic analysis of these enzymes in order to understand their phylogenetic relationships. Our data unveiled four distinct clades of the EndoIII enzymes, EndoIII group 1, 2, 3 and 4, of which the DrEndoIII homologs (DrEndoIII1, 2 and 3) and their respective *Deinococcus* homologs, are observed in EndoIII groups 1, 2 and 3 (Figure 1). Group 4 contained mammalian, higher eukaryote and archea EndoIII homologs which were included as outgroups to the *Deinococcus* EndoIII enzymes.

Within the EndoIII group 1, 2 and 3, it is possible to observe that the EndoIII group 1 and 3 are more closely related to each other than to the EndoIII group 2. It also appears that DrEndoIII1, 2 and 3 are most closely related to their respective EndoIII homologs from *D. wulumuqiensis*. It can also be observed that both DrEndoIII1 and DrEndoIII2 are least related to the homologous EndoIII enzymes from *D. peraridilitoris*, which does not have a DrEndoIII3 homolog. Furthermore, DrEndoIII2 from the EndoIII group 2 is most closely related to bacteria, and actinobacteria such as *Bacillus subtilis*, *E.coli*, *Mycobacterium tuberculosis* and to the desiccation resistant extremophile cyanobacteria *Chroococcidiopsis thermalis*. Regarding DrEndoIII1 and DrEndoIII3, our data suggest that these enzymes are more related to archaea due to the clade formation of both groups with the hyperthermophilic archeum *Pyrobaculum aerophilum.* These phylogenetic relations are in agreement with what was described previously (Makarova et al., 2001).

Moreover, our data suggest that the enzymes within the EndoIII group 3 may not be specific for the *Deinococcus* genus as one of the *Arabidopsis thaliana* EndoIII enzymes (F4JCQ3) is included in this clade. However, a more in depth analysis has to be performed regarding this proposition as the bootstrap value of this relation is 45 in our study. The second *A. thaliana* EndoIII (B9DFZ0) can be found in the same clade as human EndoIII (hNTH1) along with the EndoIII homologs from *Sacharomyces cerevisae* in EndoIII group 4 (Figure 1). This clade, like the EndoIII group 1 and 3, revealed to be related to extremophilic archaea organisms such as *Archaeoglobus fulgidus* and *Thermococcus gammatolerans*.

### 3.2. DrEndoIII KO mutants are not sensitive to γ- or UV-C radiation

**Figure 2:**
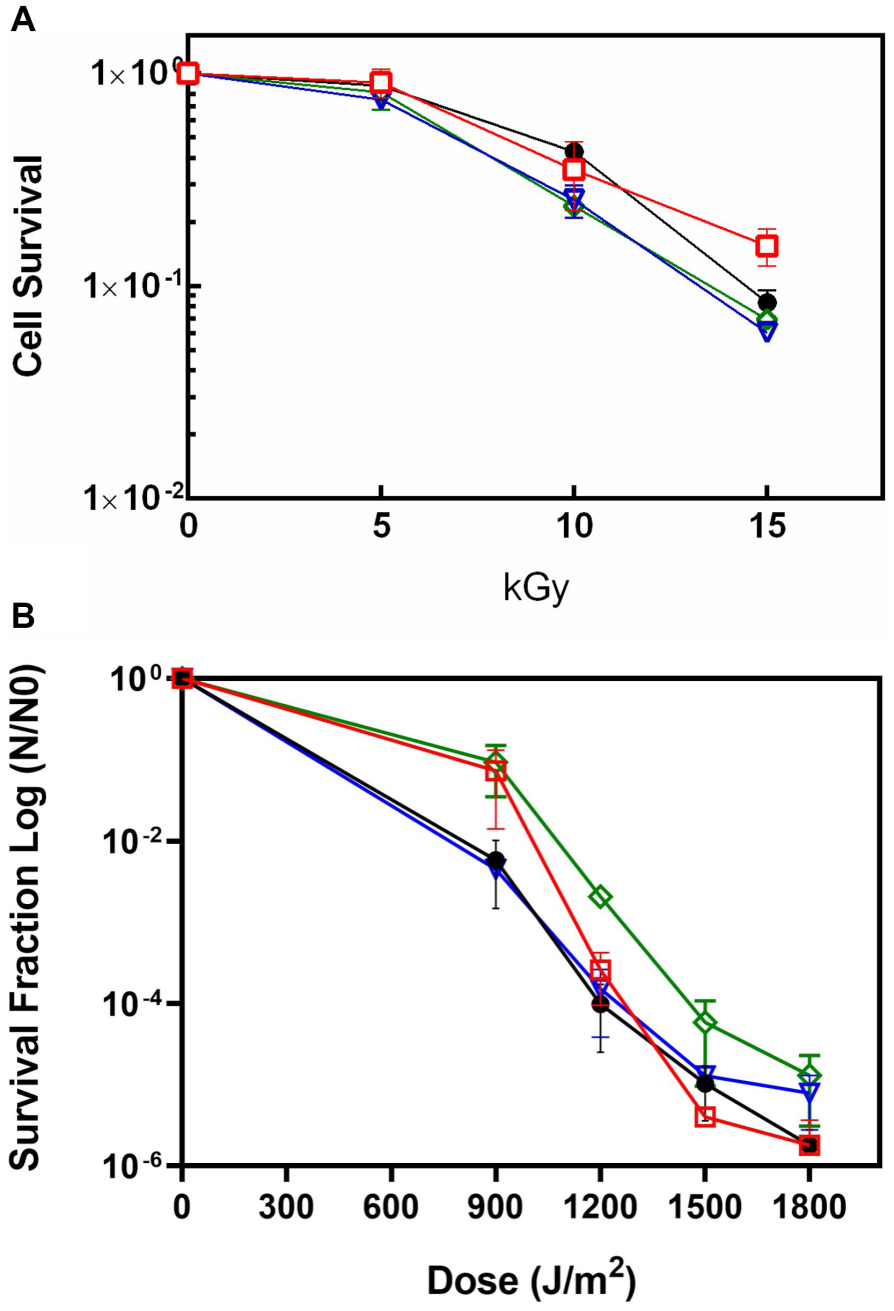
Survival rate of WT (●), ΔEndoIII1 (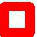), ΔEndoIII2 (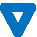) and ΔEndoIII3 (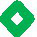) strains upon exposure to ionizing irradiation **(A)** and UV-C irradiation **(B)**. Experimental data from one out of three replicates of all the knockout mutants are shown in Supplementary figure S1.

In order to determine the role of the DrEndoIII enzymes for radiation resistance of *D. radiodurans*, the KO strains ΔEndoIII1, ΔEndoIII2, and ΔEndoIII3 as well as the WT were exposed to increasing doses of both γ- and UV-C radiation followed by an analysis of their cell survival rates. The results of these experiments revealed no major differences in the sensitivity of the three mutants when exposed to γ-radiation, even up to a dose of 15 kGy (Figure 2A). Regarding the UV-C radiation, we observed that the sensitivities of the KO strains are similar to the WT, except for at 900 J/m^2^ where ∆EndoIII1 and ∆EndoIII3 are less sensitive than the WT and ∆EndoIII2 (Figure 2B and Supplementary Figure S1).

### 3.3. DrEndoIII KO mutants are not sensitive to H_2_O_2_ but more resistant to MV than the WT

**Figure 3:**
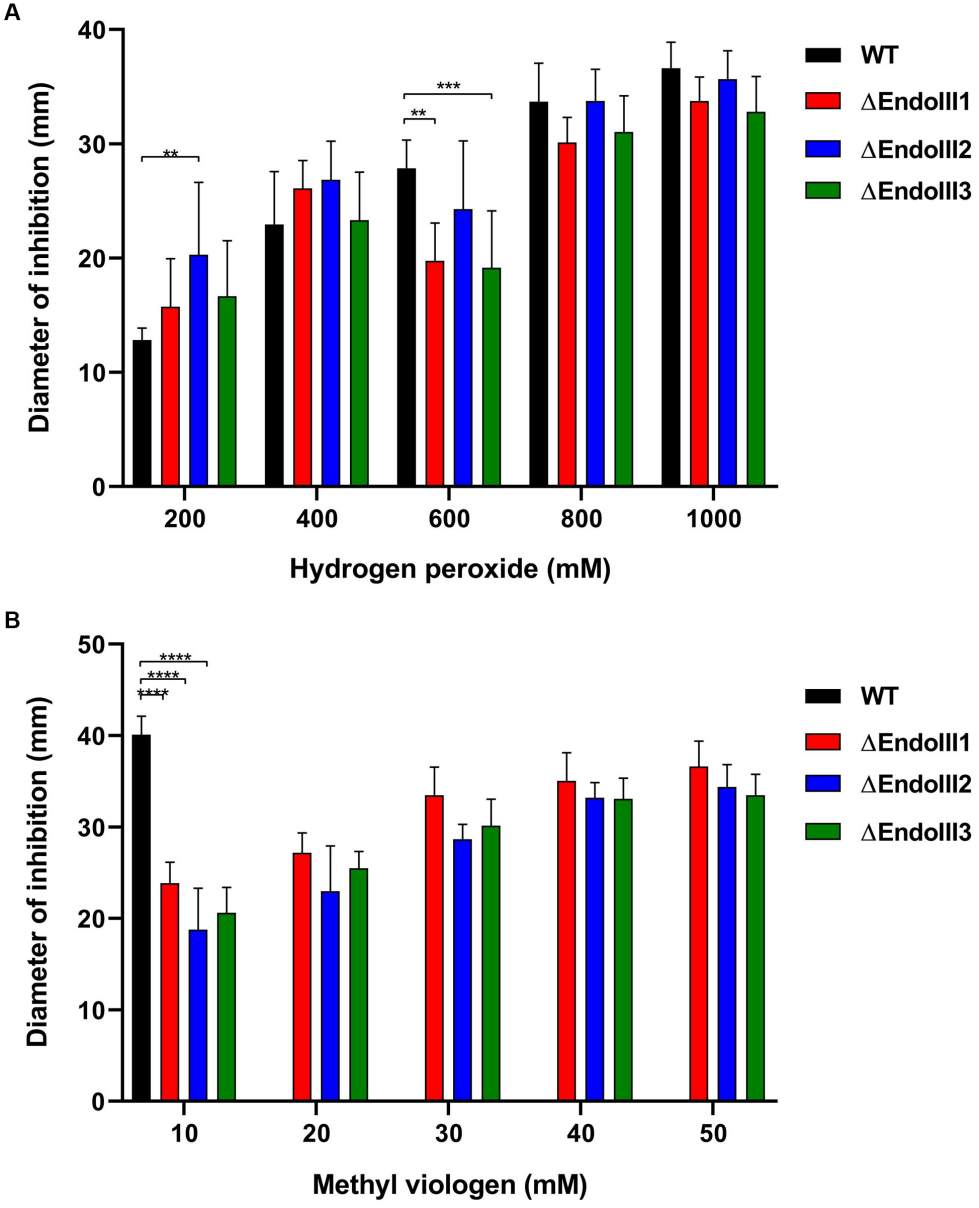
Sensitivity assays to H_2_O_2_ **(A)** and MV **(B)** of WT and ∆EndoIII1, ∆EndoIII2 and ∆EndoIII3. Data for the WT is not included in the graph for MV concentrations higher than 10 mM because the diameter of inhibition had reached the limit of the plate and was thus not possible to measure at this point. The statistic test of three replicates was done with a Two-Way ANOVA and the pvalues are represented following the GP style: 0.1234 (ns), 0.0332 (^*^), 0.0021 (^**^), 0.0002 (***), <0.0001 (^****^).

The role of the three DrEndoIII homologs for oxidative stress resistance of *D. radiodurans* was analysed by growing the KO strains on solid media in presence of the oxidative stress agents H_2_O_2_ and MV. Regarding the H_2_O_2_ assay, the KO strains displayed the same resistance as the WT except at 200 mM where ∆EndoIII2 is more sensitive than the WT and at 600 mM where ∆EndoIII1 and ∆EndoIII3 are slightly more sensitive than the WT (Figure 3A). We suggest that this is caused by the WT being resistant to H_2_O_2_ at low concentrations, while it becomes more susceptible to stress at higher concentrations.

Regarding the MV assays, the ∆EndoIII strains presented a significant increase in resistance compared to the WT (Figure 3B). At a concentration of 10 mM MV, the diameter of inhibition for the WT was double the size of the mutants. At concentrations higher than 10 mM the inhibition zone of the WT extended the size of the plate, while it was still possible to measure the inhibition zone of the ΔEndoIII strains. It was thus not possible to compare the resistance between the WT and the mutants at these concentrations. Among the KO strains there were no significant differences in their sensitivity to MV at concentrations higher than 10 mM.

### 3.4. Effects of MV-induced oxidative stress during cell growth

Based on the observation that the KO strains were more resistant to MV than the WT we decided to perform detailed growth studies of them in presence of this stress agent. In these experiments the growth of *D. radiodurans* KO and WT strains in liquid TGY medium were monitored in absence and presence of MV (0.1 mM).

**Figure 4:**
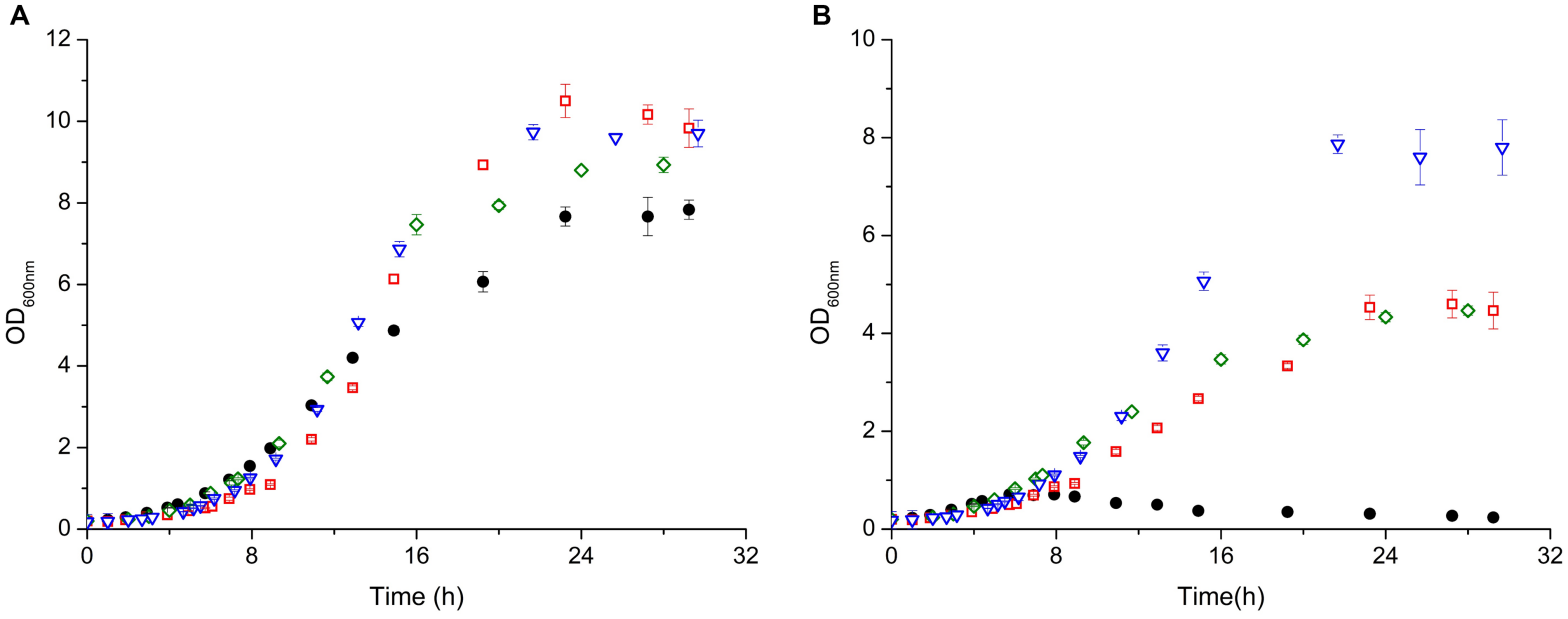
Growth curves of *D. radiodurans* (WT (●), ΔEndoIII1 (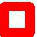), ΔEndoIII2 (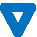) and ΔEndoIII3 (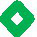)) under control conditions **(A)** and in the presence of 0.1 mM MV **(B)**. Growth rates from each strain were calculated in the exponential phase (Supplementary Table S1).

The results showed that the WT and the KO strains grow similarly under normal conditions for about 8 hours. After this point the growth starts diverting, which is reflected in different growth rates (calculated from the exponential phase - Supplementary Table S1) and with all the strains reaching different final optical densities (OD_600nm_) ranging from 8 to 9.7 (Figure 4). The WT and ΔEndoIII3 were the strains which reached the lowest final OD_600_ (8.0 and 8.5, respectively), while ΔEndoIII1 and ΔEndoIII2 reached the highest cell densities (OD_600_ = 9.7) under these conditions (Figure 4; Supplementary Table S1). All strains reached the stationary phase at around 16–20 h after inoculation (Figure 4).

In the presence of MV the growth of all the strains was affected. The WT was most affected and only able to grow for one generation (OD_600nm_ ~ 0.6) before entering a lag phase as previously observed (Santos et al., 2019) (Figure 4B). Compared to the WT (Figure 4A) the three KO strains seem to be less sensitive to the stress induced by MV, being able to grow to final OD_600nm_ ≥ 4 (Figure 4B). From all the KO strains, ΔEndoIII1 revealed to be most affected under oxidative stress with the highest difference in terms of final OD_600nm_ in stressed compared to unstressed condition (∆OD_600nm_ = 5.3) and growth rate (8.13%) (Figure 4; Supplementary Table S1). Interestingly, the least sensitive strain was ΔEndoIII2, which demonstrated lowest differences in final OD_600nm_ in stress compared to no stress condition (∆OD_600nm_ = 1.8). Simultaneously, it was the second most affected strain in terms of growth rate of all the KO strains (1.52%) (Figure 4; Supplementary Table S1). ΔEndoIII3, like ΔEndoIII1, was more affected to the stress agent than ΔEndoIII2 in terms of final OD_600nm_ differences (∆OD_600nm_ = 4.44), however it revealed to be the least affected in terms of growth rates differences (0.83%) (Figure 4; Supplementary Table S1).

### 3.5. Expression profiling of e*ndoIII* genes under MV-induced stress

The observation that the ΔEndoIII2 KO strain reached a two times higher OD_600_ than the ΔEndoIII1 and ΔEndoIII3 strains under oxidative stress conditions, suggested that the lack of one gene encoding for a DrEndoIII enzyme might be compensated for by overexpression of the other two *endoIII* genes in this condition. In order to investigate this hypothesis an expression profiling analysis of the genes encoding for DrEndoIII1, DrEndoIII2 and DrEndoIII3 (*endoIII1*, *endoIII2* and *endoIII3,* respectively) was performed in *D. radiodurans* KO and WT strains in both normal and stressed conditions. Cells from each strain were collected at different time points (0, 2 and 5 h) under both conditions during cell growth and were used for total RNA extraction and cDNA synthesis followed by RT-PCR. The MV concentration in the stressed conditions was 0.1 mM. Two housekeeping genes (*GAPDH* and *gyrA*) were used for normalization of the gene expression levels.

**Figure 5:**
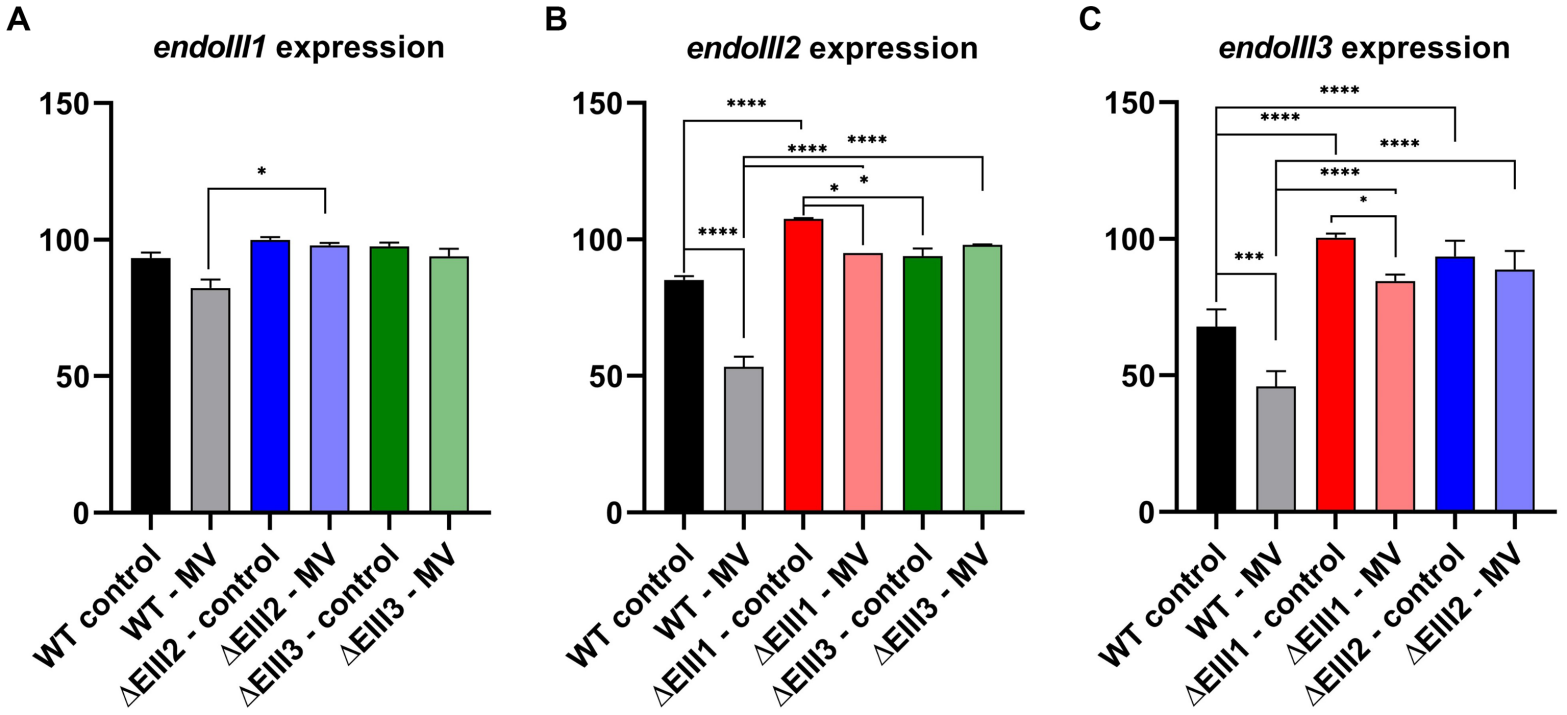
Gene expression analysis of *endoIII1* **(A)**, *endoIII2* **(B)** and *endoIII3* **(C)** in *D.radiodurans* strains (WT, ΔEndoIII1, ΔEndoIII2, ΔEndoIII3). Two growth conditions were analyzed (control and 0.1 mM MV). The data was normalized using housekeeping genes (*GAPDH* and *gyrA*) which correspond to 100% expression level. Two-Way ANOVA tests were performed to determine significant differences (*p* value under GP style: 0.1234 (ns), 0.0332 (^*^), 0.0021 (^**^), 0.0002 (^***^), <0.0001 (^****^)). Gene expression levels presented are for the last time point (5 h) since no significant differences were observed between the different time points (Supplementary Figure S3).

Overall, one of the most noticeable results of this experiment is that the expression level of *endoIII3* is lower than of both *endoIII1* and *endoIII2* in the WT under normal conditions. We also observed that in the stressed condition the expression of *endoIII2* and *endoIII3* in the WT were significantly reduced (~50%) while the *endoIII1* expression was not significantly affected (Figures 5A–C; Supplementary Figure S2).

An analysis of the expression levels of *endoIII1* in the WT, ΔEndoIII2 and ΔEndoIII3 in normal and stressed conditions (Figure 5A), revealed that this gene is expressed at similar levels in all the strains in both conditions. Only in ΔEndoIII2 it is possible to observe a significant increase (16%) of the *endoIII1* expression level under stressed condition, compared to the WT (Figure 5A).

Furthermore, the expression levels of *endoIII2* in the WT and ΔEndoIII1 and ΔEndoIII3 (Figure 5B) in unstressed and stressed conditions are higher in the KO strains than in the WT in both conditions. Under normal conditions, we observed a significant increase (23%) in the *endoIII2* expression level in the ΔEndoIII1 strain compared to the WT, being overexpressed when compared with the housekeeping gene (> 100%). The increase in the expression level of *endoIII2* in ΔEndoIII3 was lower than in the WT (14%) in normal conditions. Furthermore, in stressed conditions a significant increase in the expression level of e*ndoIII2* to 42 and 45% was observed in both ΔEndoIII1 and ΔEndoIII3, respectively, when compared to the WT. Under the same condition a 32 and 13% decrease in the level of *endoIII2* expression was observed in the WT and ΔEndoIII1, respectively, when compared to normal conditions (Figure 5B).

Finally, the expression levels of *endoIII3* were higher in ΔEndoIII1 and ΔEndoIII2 compared to the WT in both growth conditions (Figure 5C). In normal conditions an increase in expression of 33 and 26% of *endoIII3* was observed in ΔEndoIII1 and ΔEndoIII2 when compared with WT. We noticed also that the level of expression of e*ndoIII3* in the ΔEndoIII1 is similar to the housekeeping genes (~100%). The expression level of *endoIII3* is decreased in both the WT and ΔEndoIII1 by 22 and 16%, respectively, under stressed compared to normal conditions. Under stress conditions an increase in the e*ndoIII3* expression level is observed for both ΔEndoIII1 (39%) and ΔEndoIII2 (43%) compared to the WT (Figure 5C).

## 4. Discussion

Based on more available genome sequenced *Deinococcus* strains, we started this work by performing a phylogenetic analysis of the three Endonuclease III enzymes among these strains. Based on an analysis of 17 fully genome sequenced *Deinococcus* species, our data suggests that DrEndoIII1 and DrEndoIII3 are archaeal type enzymes as previously suggested (Makarova et al., 2001). We could also hypothesize that DrEndoIII3 may not be a specific variant for the *Deinococcus* species, however this requires further analysis and validation (Figure 1). Regarding DrEndoIII2 our data suggest that it is more closely related to bacteria than to yeast.

The survival of the ∆EndoIII strains upon exposure to IR revealed no significant differences when compared to the WT (Figure 2A). This was surprising, especially in the case of ΔEndoIII2, since DrEndoIII2 is the apparent main EndoIII enzyme in *D. radiodurans* (Sarre et al., 2019). However, similar results from other IR experiments have been observed previously (Cunningham and Weiss, 1985; Hua et al., 2012), even in the case of multiple KO strains. In these cases, it was suggested that other repair pathways and enzymes from the BER pathway are able to repair the lesions that are introduced into the DNA under these conditions. It is likely that this is the case also for our experiment, knowing that IR causes both direct and indirect damage to DNA, leading to base modifications (e.g., 8-oxoG and Tg) and strand breaks which can be dealt with both in BER and homologous recombination (Cox and Battista, 2005; Slade and Radman, 2011; Krokan and Bjørås, 2013; Dizdaroglu, 2015; Timmins and Moe, 2016). We can also not exclude the possibility that the ROS scavenging system (e.g., catalases and SOD) and other irradiation protection mechanisms (e.g., high level of pigments in the membrane) is protecting *D. radiodurans* from severe damage in this case.

Exposure of our KO strains to UV-C radiation revealed no observable response and resistance differences to *D. radiodurans* WT (Figure 2B). The most abundant DNA lesions generated by UV-C radiation, are the mutagenic and cytotoxic cyclobutane pyrimidine dimers (CPDs) and pyrimidine–pyrimidone (6-4) photoproducts (6–4PPs) (Battista, 1997; Sinha and Häder, 2002; Slade and Radman, 2011; Timmins and Moe, 2016), which are repaired via the NER and the UV damage endonuclease (UVDE) pathways (Tanaka et al., 2005; Blasius et al., 2008; Slade and Radman, 2011; Timmins and Moe, 2016). UV radiation also generates other photoproducts and lesions normally associated with oxidative damages in DNA such as pyrimidine hydrate, Tg, dipurine adducts, ROS (mainly singlet oxygen (^1^O_2_), protein oxidation and 8-oxoG) (Demple and Linn, 1982; Zhang et al., 1997; Friedberg et al., 2006; Krisko and Radman, 2010; Chatterjee and Walker, 2017). Thus, we suggest that the UV-damages generated in this experiment might have been repaired in the NER and UVDE pathways and that the remaining oxidative damages might have been efficiently repaired by the BER DNA glycosylases (8-oxoG DNA glycosylase and the EndoIII’s) even in the absence of one of the DrEndoIII enzymes.

In order to analyze the response of the *D. radiodurans* strains to oxidative stress, they were exposed to either H_2_O_2_ or MV (Figure 3). Both agents are responsible for damage of nucleobases, nucleotides (oxidized pyrimidines and purines (eg. Tg and 8 oxo-G)) (Demple and Linn, 1982; Petrovská and Dušinská, 1999; Slade and Radman, 2011), and proteins containing iron–sulfur and heme groups (Munteanu et al., 2015). Superoxide radicals can be converted to H_2_O_2_ and consequently to Hydroxyl radicals leading to major DNA modifications (Maynard et al., 2009; Dizdaroglu, 2015; Gonzalez-Hunt et al., 2018). Both H_2_O_2_ and superoxide radicals are generally detoxified by different scavengers systems such as superoxide dismutase, catalases and Mn complexes (Culotta and Daly, 2013). Here, exposure of the KO and WT strains to H_2_O_2_ demonstrated that they possessed similar sensitivity to this oxidation damaging agent (Figure 3A). This response have also been observed previously of other *D. radiodurans* and *E. coli* EndoIII KO mutants (Cunningham and Weiss, 1985; Hua et al., 2012).

Regarding the MV stress experiments, our data clearly revealed an effect on the growth demonstrating a reduction in both growth rate and final OD on all strains tested. As previously observed, the growth of the WT was severely affected, leading to a decrease of the growth before entering a lag phase (Santos et al., 2019) (Figure 4B). The three DrEndoIII KO strains proved to be more resistant being able to grow to a higher final optical density upon addition of the damaging agent than the WT (> 17x). Out of the three KO strains, ∆EndoIII1 was most severely affected by the addition of MV, reducing the final OD_600_ from 10 to almost 4 and a reduction in the growth rate from 0.2563 to 0.175 h^−1^ (Figure 4B; Supplementary Table S1). ∆EndoIII2 showed higher resistance levels than the other mutants (Figure 4B). This was not expected since DrEndoIII2 is considered the main EndoIII enzyme responsible for repair of oxidation damaged pyrimidines in DNA (Sarre et al., 2019). Although ΔEndoIII3 unveiled to be the strain with the lowest growth rate under normal conditions it was also the strain with the smallest difference in growth rates when comparing both growth conditions (Supplementary Table S1). This result suggests that DrEndoIII3, in general, is important for oxidative stress resistance in *D. radiodurans* despite its lack of classic EndoIII substrate specificity (Sarre et al., 2019).

A gene expression analysis was carried out to evaluate the importance of the three DrEndoIII homologs in oxidative stress resistance (MV) at the gene level (Figure 5). The results revealed that *endoIII1* is expressed at similar levels in all strains and conditions suggesting that it is important during cell growth (Figure 5A). These results do not correspond to findings in other transcriptome experiments where irradiation was used to induce oxidative stress. In one case it was shown that the expression levels of *endoIII1* were down regulated upon exposure to 15 kGy of IR compared to control conditions (Liu et al., 2003). In the other case it was shown that *endoIII1* was DNA-damage inducible immediately upon exposure to 2 kGy of IR (Chen et al., 2007). However, the stress induction in both cases is different and is thus not directly comparable to our experiment. Even so, it indicates that the level of expression may be different depending on the damage that is inflicted into the DNA. In our case, a generally high expression level of *endoIII1* was observed in both normal and stressed condition in WT as well as KO strains, indicating a generally high importance of this protein during cell growth.

Opposite to *endoIII1*, both *endoIII2* and *endoIII3* genes are upregulated in the KO strains under oxidative stress conditions compared to the WT (Figures 5B,C). We even observed that *endoIII2* is overexpressed surpassing the expression of the housekeeping genes when *endoIII1* is knocked out (ΔEndoIII1) under normal conditions and that *endoIII3* reaches the expression levels of the housekeeping genes. This is also in contrast to what has been reported previously. In these studies the level of expression of the *endoIII2* and *endoIII3* genes did not change (Liu et al., 2003), and the *endoIII2* gene was not significantly induced upon IR exposure (Chen et al., 2007). Again, the stress induction in those studies is not directly comparable to our study, and we can only assume that the level of damage induced by exposure of the cells to MV is not as dramatic as for IR. However, this brings support to our hypothesis about *endoIII1* being in general very important for *D. radiodurans* in both normal and stressed conditions, suggesting that this enzyme may play additional roles in the cells which goes beyond DNA repair. This corresponds well to our knowledge that DrEndoIII1 possesses specificity for oxidation damages in single stranded DNA in addition to its primary function as a DNA glycosylase in the presence of Tg in double stranded DNA substrates (Sarre et al., 2019). Multiple roles of DNA glycosylases have been observed previously, e.g., of human uracil DNA N-glycosylases (UNG) which is involved in both BER and replication (Krokan et al., 2002). Due to its activity on single stranded DNA, it is thus not unlikely that DrEndoIII1 is involved in DNA replication repair, however, this will have to be explored in future experiments.

## 5. Conclusion

Here we have provided further insights into the role/importance of the three Endonuclease III enzymes in *D. radiodurans* when this bacterium is exposed to oxidative stress. Despite the lack of observed *in vitro* activity/function for DrEndoIII3 from previous studies, our data suggests that this gene product is extremely important for the bacteriums’ oxidation damage resistance. This is noticeable in all the KO strains, with observed upregulation of *endoIII3* in both ΔEndoIII2 and ΔEndoIII1 KO strains. Also, in ΔEndoIII2 the overexpression of *endoIII3* coupled with *endoIII1* was revealed to be the best combination to cope with oxidative stress and is a good example of how the lack of one of the *endoIII* genes may be compensated for by overexpression of the other two. Moreover, the observation that the expression of *endoIII1* is important in all strains in both normal and stressed conditions, suggests that this enzyme may play multiple roles in *D. radiodurans*. The role of both DrEndoIII1 and DrEndoIII3 will therefore be further explored in future studies. Thus, we conclude that all three DrEndoIII enzymes are important in the resistance mechanisms of *D. radiodurans* towards oxidative stress, however, the contribution of the individual enzymes in this process is yet to be fully disclosed. One possible approach to reveal new information in this regard is to perform a comparative gene clustering analysis of each EndoIII group using available genome data of sequenced *Deinococcus* strains.

## Data availability statement

The original contributions presented in the study are included in the article/Supplementary material, further inquiries can be directed to the corresponding author.

## Author contributions

FR: Investigation, Methodology, Visualization, Writing – original draft, Formal analysis. GM: Investigation, Visualization, Writing – review & editing, Formal analysis. AG: Writing – review & editing, Methodology, Supervision, Formal analysis. SI: Investigation, Writing – review & editing. PS: Methodology, Writing – review & editing, Supervision, Visualization. CR: Supervision, Writing – review & editing, Conceptualization, Funding acquisition. EM: Conceptualization, Funding acquisition, Supervision, Writing – review & editing, Project administration.

## Funding

The author(s) declare financial support was received for the research, authorship, and/or publication of this article. This work was supported by FCT—Fundação para a Ciência e a Tecnologia, I.P., through MOSTMICRO-ITQB R&D Unit (UIDB/04612/2020, UIDP/04612/2020) and LS4FUTURE Associated Laboratory (LA/P/0087/2020), Centre National de la Recherche Scientifique and the University Paris-Saclay, research projects PTDC/QUI/BIQ/100007/2008, PTDC/BBBBEP/0561/2014, PTDC/BIA-BFS/31026/2017, PTDC/BIA-BQM/31317/2017, post doc fellowship SFRH/BPD/97493/2013 (EM), PhD fellowships SFRH/BD/132966/2017 and COVID/BD/152598/2022 (FR) and SFRH/BD/06723/2020 (AGG). Funding is also acknowledged for the TIMB3 and IMpaCT project, European Union’s Horizon 2020 research and innovation program, under grant agreement No 810856 and No 857203, respectively, and the Research Council of Norway, project number: 183626.

## Conflict of interest

The authors declare that the research was conducted in the absence of any commercial or financial relationships that could be construed as a potential conflict of interest.

## Publisher’s note

All claims expressed in this article are solely those of the authors and do not necessarily represent those of their affiliated organizations, or those of the publisher, the editors and the reviewers. Any product that may be evaluated in this article, or claim that may be made by its manufacturer, is not guaranteed or endorsed by the publisher.
