# Supplementary material for: Insights into the role of three Endonuclease III enzymes for oxidative stress resistance in the extremely radiation resistant bacterium Deinococcus radiodurans
Source: Front Microbiol. 2023 Sep 12;14:1266785. doi: 10.3389/fmicb.2023.1266785 (PMC10523315; doi:10.3389/fmicb.2023.1266785)
Supplement: Supplementary file 1 [file Data_Sheet_1.docx]

**Supplementary information**

Insights into the role of three Endonuclease III enzymes for oxidative stress resistance in the extremely radiation resistant bacterium *Deinococcus radiodurans*

Filipe Rollo^1^, Guilherme D. Martins^1^, André G. Gouveia^1^, Solenne Ithurbide^2,3^, Pascale Servant^2^, Célia V. Romão^1^ and Elin Moe^1,4^*

^1^ Instituto de Tecnologia Química e Biológica António Xavier, Universidade NOVA de Lisboa, Av. Da República, 2780-157, Oeiras, Portugal

^2^ Université Paris-Saclay, CEA, CNRS, Institute for Integrative Biology of the Cell (I2BC), 91198, Gif sur Yvette , France

- ^3^ Present address: Department of Microbiology, Infectious Diseases and Immunology, Faculty of Medicine, University of Montreal, Canada

^4^ Department of Chemistry, UiT – the Arctic University of Norway, Tromsø, Norway.

* Corresponding author: Elin Moe ([elinmoe@itqb.unl.pt](mailto:elinmoe@itqb.unl.pt) and elin.moe@uit.no)

**Supplementary Table**

Table S1. Growth rates and comparison of differences in growth rates and final OD_600nm_ from each *D. radiodurans* strain (WT, ∆EndoIII1, ∆EndoIII2 and ∆EndoIII3) growth under normal and MV-induced conditions. Growth rates were calculated from the exponential phase with an R^2^ ≥ 0.993, by using Growth rate = ((ln(OD_f_-OD_i_))/tf-ti). The ΔOD_600_ is a measurement of final OD between unstressed and stressed conditions.

|  | WT | WT MV | ∆EndoIII1 | ∆EndoIII1 MV | ∆EndoIII2 | ∆EndoIII2 MV | ∆EndoIII3 | ∆EndoIII3 MV |
| --- | --- | --- | --- | --- | --- | --- | --- | --- |
| Growth rate | 0.1736± 0.0006 | n.a. | 0.2563 ± 0.0102 | 0.175 ± 0.0039 | 0.2126 ± 0.0074 | 0.1974 ± 0.0058 | 0.1903 ± 0.0055 | 0.182 ± 0.0049 |
| Growth rate difference | n.a. | | 0.0813 | | 0.0152 | | 0.0083 | |
| ΔOD_600nm_ | 7.74 | | 5.3 | | 1.8 | | 4.44 | |

**Supplementary Figures**

**
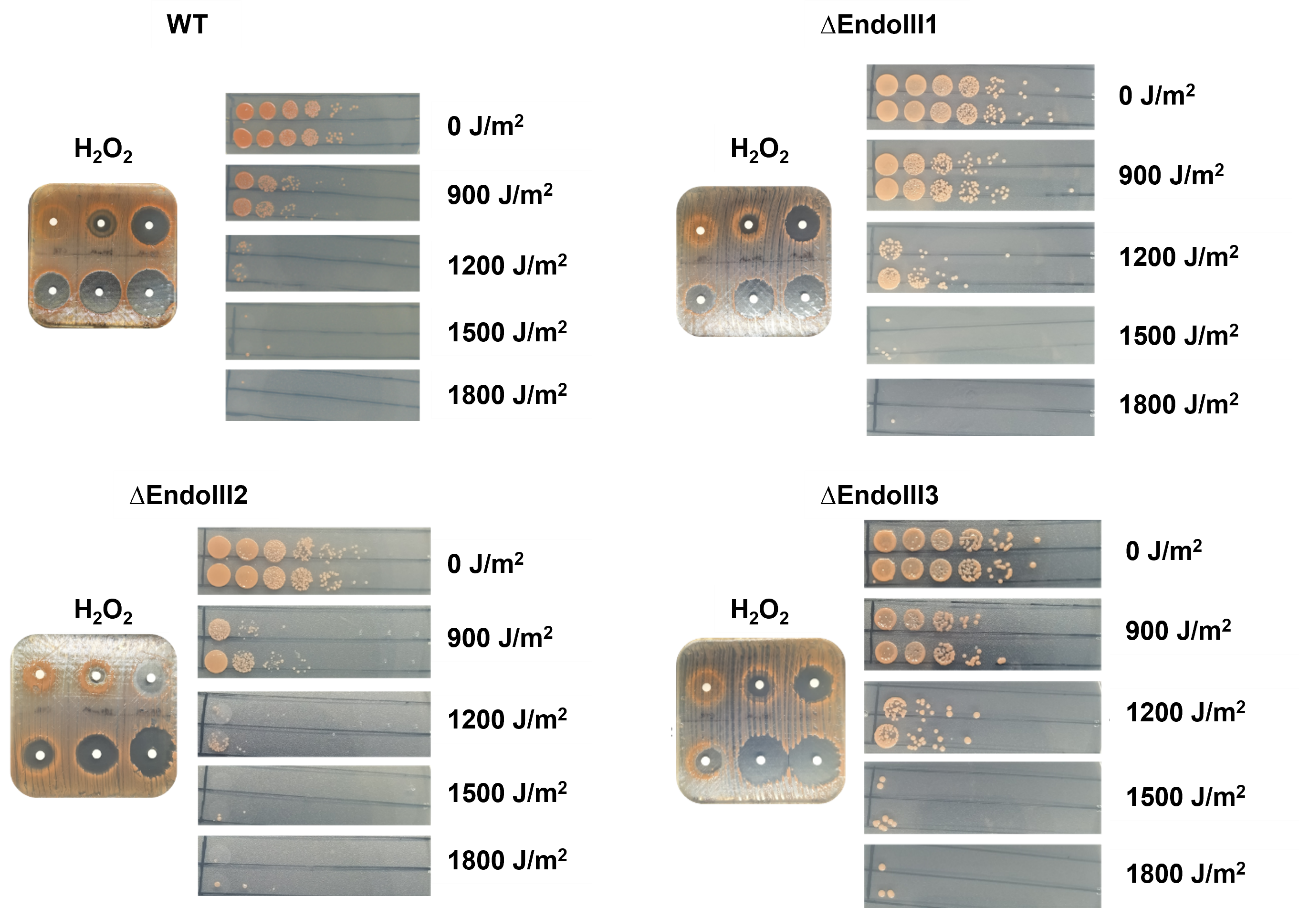
**

**Supplementary figure S1.** - Results from one of the replicates, from the H_2_O_2_ and UV-C assays, TGY of the WT, ∆EndoIII1, ∆EndoIII2 and ∆EndoIII3 assays. In the H2O2 stress assays, triplicates were done and, in the UV-C assays, two replicates in the same plate were used as well as two plate replicates. Both types of assays had two biological replicates.

**
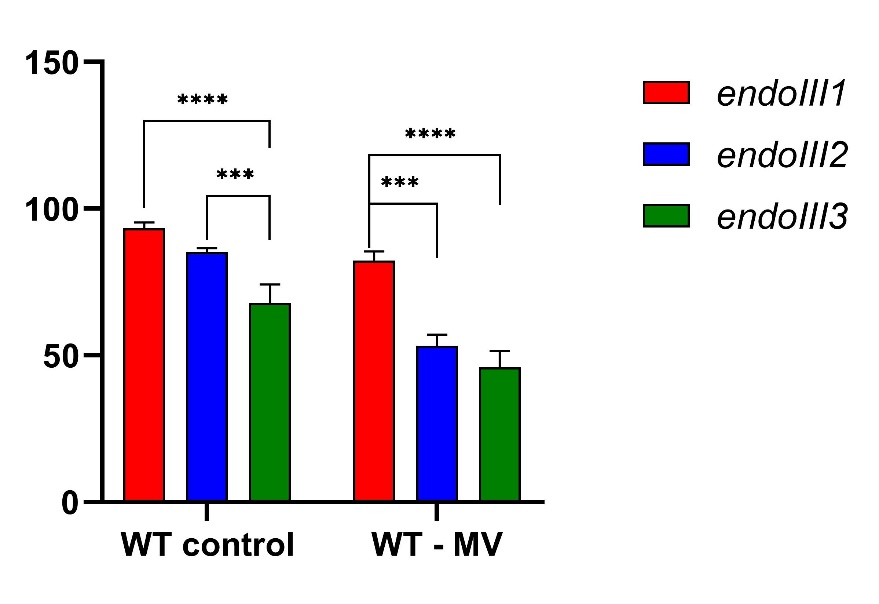
**

**Supplementary figure S2.** Gene expression analysis of *endoIII1*, *endoIII2* and *endoIII3* in *D. radiodurans* WT. Two growth conditions were analyzed (control and 0.1 mM MV). The data was normalized using housekeeping genes (*GAPDH* and *gyrA*) which correspond to 100% expression level. Two-Way ANOVA tests were performed to determine significant differences (pvalue under GP style: 0.1234 (ns), 0.0332 (*), 0.0021 (**), 0.0002 (***), <0.0001 (****)).

**
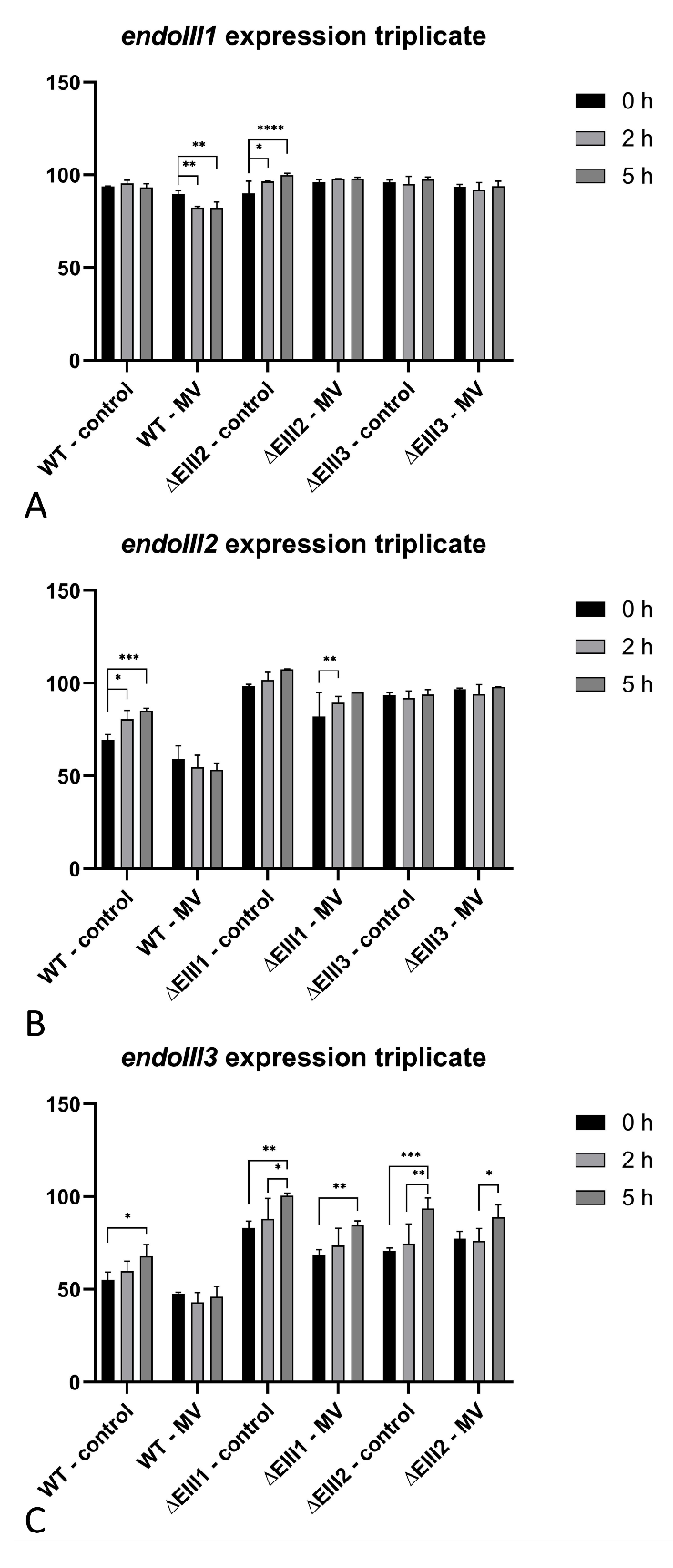
**

**Supplementary figure S3.** Gene expression analysis of *endoIII1* (A), *endoIII2* (B) and *endoIII3* (C) in *D. radiodurans* strains (wild-type, ΔEndoIII1, ΔEndoIII2, ΔEndoIII3). Two growth conditions (control and methyl viologen (MV) and three different time points (0h, 2h and 5h) were analyzed. The data was normalized using housekeeping genes (*GAPDH* and *gyrA*) which correspond to 100% expression level. Two-Way ANOVA tests were performed to determine significant differences between each time point within each growth condition (pvalue under GP style: 0.1234 (ns), 0.0332 (*), 0.0021 (**), 0.0002 (***), <0.0001 (****)).
